# Small and Simple, yet Sturdy: Conformationally Constrained Peptides with Remarkable Properties

**DOI:** 10.3390/ijms22041611

**Published:** 2021-02-05

**Authors:** Krištof Bozovičar, Tomaž Bratkovič

**Affiliations:** Department of Pharmaceutical Biology, Faculty of Pharmacy, University of Ljubljana, Aškerčeva 7, SI-1000 Ljubljana, Slovenia; kristof.bozovicar@ffa.uni-lj.si

**Keywords:** constrained peptides, macrocycles, crosslinking, enzymatic cyclization, stapling, hairpin loop

## Abstract

The sheer size and vast chemical space (i.e., diverse repertoire and spatial distribution of functional groups) underlie peptides’ ability to engage in specific interactions with targets of various structures. However, the inherent flexibility of the peptide chain negatively affects binding affinity and metabolic stability, thereby severely limiting the use of peptides as medicines. Imposing conformational constraints to the peptide chain offers to solve these problems but typically requires laborious structure optimization. Alternatively, libraries of constrained peptides with randomized modules can be screened for specific functions. Here, we present the properties of conformationally constrained peptides and review rigidification chemistries/strategies, as well as synthetic and enzymatic methods of producing macrocyclic peptides. Furthermore, we discuss the in vitro molecular evolution methods for the development of constrained peptides with pre-defined functions. Finally, we briefly present applications of selected constrained peptides to illustrate their exceptional properties as drug candidates, molecular recognition probes, and minimalist catalysts.

## 1. Introduction

With the permutation of a limited set of residues, natural peptides can display remarkable structural diversity. The incorporation of unnatural and *D*-amino acids in synthetic peptides further widens their chemical structure space. This feature is increasingly being harnessed by medicinal chemists to pursue selective and potent pharmacological agents, where peptides are either used as pharmaceuticals or their pharmacophores are sampled to design peptidomimetics with improved drug-like properties. The modular nature of peptides makes them amenable to combinatorial chemistry. In recent decades numerous innovative combinatorial peptide library platforms have been developed, either synthetic, biological (i.e., genetically encoded), or semisynthetic, enabling systematic high-throughput interrogation of peptides’ chemical landscape. Unfortunately, simple linear peptides are typically conformationally undefined in solution, which compromises binding affinity due to high entropic cost upon target binding [1,2]. Several strategies have been developed, however, to limit peptides’ flexibility or enforce specific secondary structural elements [3,4,5,6]. These include chemical and enzymatic cyclization to produce macrocycles (including multicyclic structures), stapling to promote helical conformations in short peptides, and β-hairpin design, both, by optimizing pairing between the antiparallel strands and turn structures. Of note, even in absence of any rational attempts to limit the flexibility of library peptides, screening campaigns have been known to result in enrichment of highly structured ligands [7,8,9,10]. This demonstrates the importance of pre-organized ligand structures for thermodynamically favored binding and/or interaction with target molecule sites inaccessible to simple linear peptides.

## 2. Properties of Conformationally Constrained Peptides

Restricting peptide conformation to the one that the ligand assumes upon target binding (or even to a subset of structures occupied by a flexible parent peptide) is highly advantageous with regard to attaining increased affinity [1]. The effect of conformational entropy on (macro)molecular interactions should not be underestimated, but at the same time structure rigidification should be meticulously optimized to allow taking on (or at least sampling of) the target-bound conformation [5,11]. Achieving the proper pre-organized fold might also lead to improved specificity because off-target binding (associated with a different conformation of the same peptide) is less likely to occur [12]. A further benefit of restricting peptide’s flexibility is to enhance its proteolytic stability, as the substrate polypeptide chain typically needs to adopt an extended conformation to be loaded in the protease active site [13].

The simplest way of constraining a peptide is to form a cyclized structure, for example, through paired cysteines, head-to-tail peptide bond, or grafting a peptide loop on a short antiparallel β-sheet motif. Although a looped peptide is typically still fairly flexible (depending on the ring size), it can only conform to a subset of structures that are accessible to its linear counterpart. ‘CLIPSing’ of linear peptides harboring cysteine residues with synthetic thiol-reactive scaffolds enables the construction of bicyclic or tricyclic structures capable of complex folds [14], functionally reminiscent of antibody paratopes. Such structures were shown to effectively bind to sites considered undruggable with conventional low-molecular-weight compounds, such as flat surfaces engaged in protein–protein interactions [15,16] and components or precursors of the bacterial cell wall [17,18]. Alternatively, the bicyclic form allows for combining two distinct functionalities in a single molecule, such as the ability to inhibit two non-homologous enzyme targets [19], or cell penetration and intracellular target binding [20,21]. Enhanced lipid membrane permeability has been reported for cationic amphipathic peptides that assume helical conformation upon interaction with cell membranes [22], and stapled and stitched (i.e., containing tandem crosslinks) peptide helices recapitulate this feature [23,24]. Cell internalization was shown to be highly dependent on the overall peptide charge, being most efficient for net charges of +3 to +5 [23]. Additionally, the stitched peptides displayed better permeability compared to the less stabilized stapled ones. The overall hydrophobic nature of the construct and the positioning of staples are also important for cell penetration (most penetrable helices contained staples at the amphipathic boundary [24]). Cell penetration seems to occur via a clathrin- and caveolin-independent, energy-dependent endocytic pathway, and likely proceeds through interaction with the negatively-charged sulfated cell surface proteoglycans [23]. Beta hairpin peptides are attractive because of their compatibility with biological library formats, and due to the vast functional diversity of this structural motif. For example, there are reports of β-hairpin peptides mimicking helical protein epitopes [25] or forming highly selective complexes with distinct precursor microRNAs [26,27].

## 3. Constrainment Strategies and Chemistries

Grafting peptides in place of surface-exposed loops of a stable protein framework ensures limited flexibility of the insert. For example, we have spliced a short peptide, known for its conformation-dependent inhibitory activity against cysteine proteases [28], between the antiparallel helices of a small scaffold protein (B domain of staphylococcal immunoglobulin-binding protein A) to design a peptide ‘aptamer’ [29]. As expected, the inhibitory activity of the looped peptide was preserved even in the presence of a reducing agent, while the disulfide-cyclized parent peptide [28] lost much of its activity as a result of linearization. Peptide aptamers, however, are typically developed through randomization of a loop sequence and the resulting library is screened for desired properties, most commonly binding affinity [30]. A similar strategy is being used in the design of artificial binding proteins where discontinuous surface-exposed epitopes of a scaffold protein, such as portions of α-helices or β-sheets, are randomized to produce a library. Both approaches have been extensively reviewed [31,32,33] and will not be discussed here further. Instead, the focus will lay on the rigidification of short linear peptides by means of (click-) chemical crosslinking and enzymatic intramolecular ligation resulting in cyclized structures. Conversely, multicomponent synthetic approaches of producing cyclic peptides [34] are challenging because the polypeptide chain needs to adopt an entropically disfavored conformation (reviewed in [35]). We also look at efforts to achieve loop formation by optimizing flanking amino acid sequences to form short but stable β-sheets, resulting in β-hairpins. Often, the different strategies are used hand in hand, for example combining enzymatic head-to-tail cyclization and chemical inter-side-chain crosslinking to produce multicyclic peptide structures, or covalent crosslinking to further stabilize peptides with a propensity of folding into β-hairpins.

### 3.1. Chemical Peptide Ligation and Bridging

**Figure 1:**
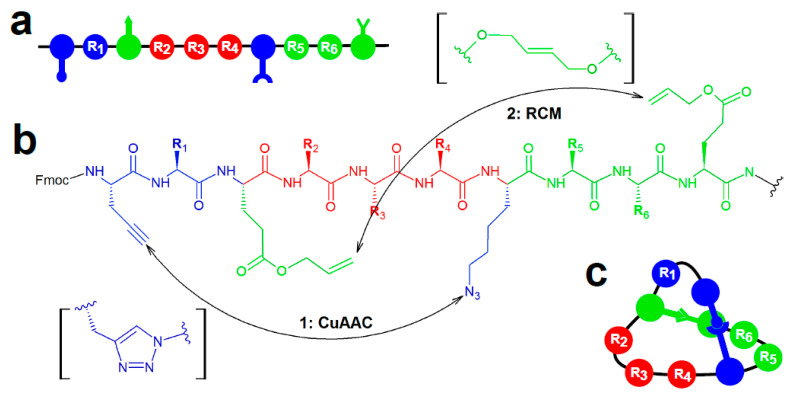
Bicyclic peptide library construction via two sequential orthologous intramolecular reactions as reported by Li et al. [36]. Schematic depiction of a library decapeptide before (**a**) and after on-resin cyclization reactions (**c**). Note that randomized residues are present in all three loops. (**b**) General structural formula of library peptides with two pairs of non-canonical residues that undergo copper(I)-catalyzed azide-alkyne cycloaddition (1: CuAAC) and ruthenium-catalyzed ring-closing metathesis (2: RCM, respectively. Moieties formed as a result of these reactions are depicted in square brackets.

During solid-phase peptide synthesis, unnatural amino acids harboring orthogonally reactive functional groups can be introduced into a polypeptide chain to selectively induce covalent crosslinks between such residues, thus forming cyclized structures. Li et al. [36] reported the construction of a bicyclic decapeptide library by combining copper(I)-catalyzed cycloaddition reaction between the N-terminal propargylglycine and the central azide lysine (position 7), and ruthenium-catalyzed alkene ring-closing metathesis reaction between the glutamate allyl esters placed centrally (position 3) and at the C-terminus (Figure 1). As these cyclization ‘handles’ were separated by stretches of randomized residues (positions 2, 4–6, 8, and 9), all three loops of the library bicycles could be considered to possess a modular structure. Importantly, such library design allowed for linearization of bicyclic structure through dealkylation and Edman degradation, required for mass spectrometry-based sequencing to identify the hits after screening.

**Figure 2:**
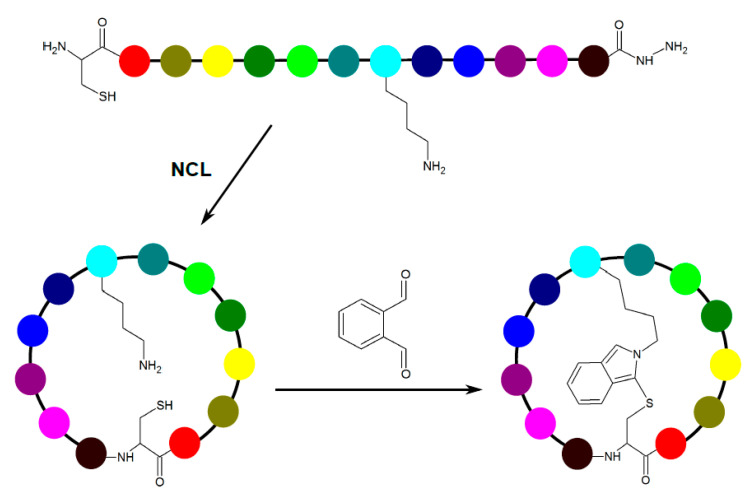
Peptide rigidification via native chemical ligation (NCL) followed by ortho-phthalaldehyde crosslinking, yielding an isoindole-bridged cyclic peptide [37].

A simple chemoselective cyclization method has recently been described by Zhang et al. [37]. Here, ortho-phthalaldehyde reacts with the amino group (lysine side chain or free N-terminus) and the cysteine’s thiol group in an unprotected linear peptide in aqueous solution, to yield an isoindole-bridged cyclic peptide. Combining the approach with hydrazide-based native chemical ligation (i.e., reaction of the N-terminal cysteine with a C-terminal hydrazide resulting in thioesterification followed by S-to-N acyl shift to form a peptide bond, freeing the thiol group [38]) rendered bicyclic peptides (Figure 2).

**Figure 3:**
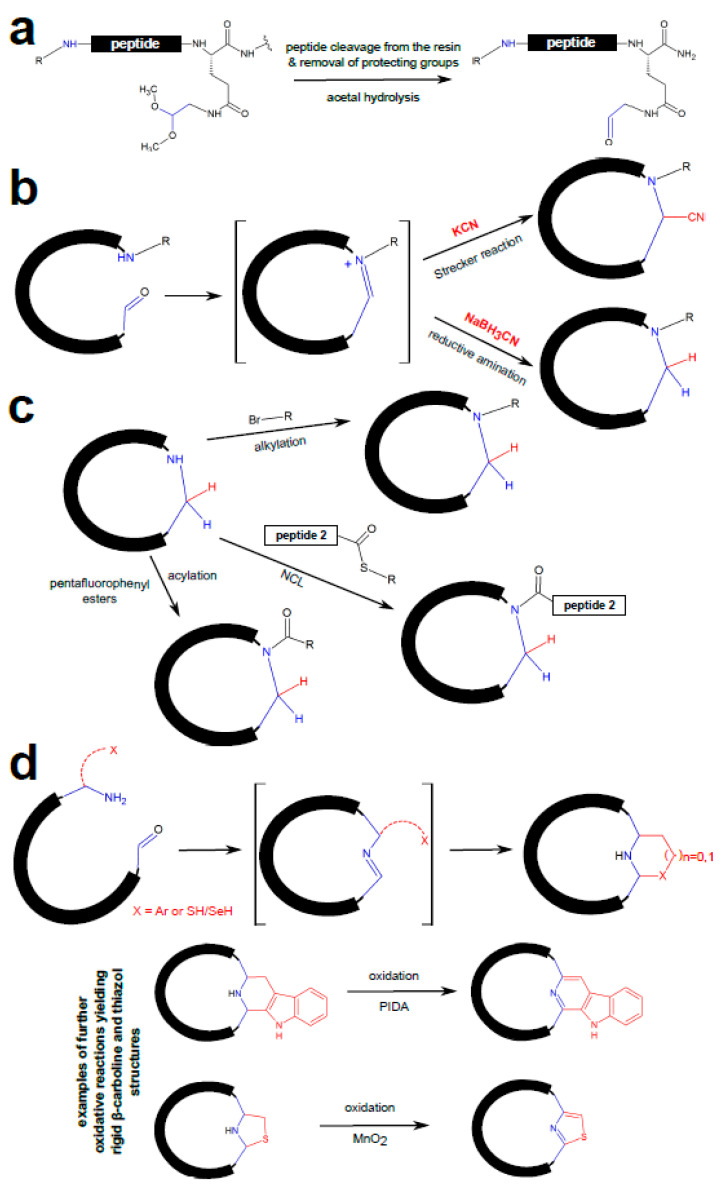
Head-to-tail macrocyclization via selective condensation of amines and aldehydes (adapted from [39]). (**a**) Incorporation of an aldehyde group at the C-terminal residue. (**b**) Use of intermolecular imine traps to prevent ring opening upon macrocyclization. (**c**) Late-stage macrocycle derivation at the site of ring closure: acylation, alkylation, and to native chemical ligation (NCL) of the resulting secondary amine. (**d**) Above: Use of intramolecular imine traps (such as aromatic rings (Ar; e.g., indoles) and thiols (SH) and selenols (SeH)) to prevent ring opening following macrocyclization. Below: Further rigidification of macrocyclices via oxidation reactions yielding aromatic ring structures. PIDA—phenyliodine(III) diacetate.

An extremely versatile synthetic route for head-to-tail macrocyclization based on selective condensation of amines and aldehydes was described by Malins et al. [39]. An aldehyde group is introduced to the C-terminal residue (e.g., in a form of aminoacetaldehyde dimethyl acetal coupled to a glutamic acid residue via delta amide linkage) during solid-phase synthesis (Figure 3a). Upon resin cleavage, the protected aldehyde group is concomitantly resolved and is free to react with the N-terminal amino group to form an imine. As this reaction is reversible, the macrocycles need to be stabilized by ‘imine traps’ (Figure 3b). Potassium cyanide proved effective as an imine trap in a Strecker reaction, allowing formation of macrocyclic α-aminonitriles. Alternatively, NaBH_3_CN was used to catalyze imine reduction. The cyclization/trapping reactions are highly tolerant to various functional groups, such as carboxylic acids, primary amides, disulfides, alcohols, phenols and imidazoles, and macrocycles of 5 to 10 residues can be prepared in high yields in aqueous solutions at room temperature even for N-alkylated amines. Furthermore, this cyclization reaction is selective for α-amino groups as unprotected internal lysine residues did not compete for the carbonyl electrophile. Finally, the authors have shown that late-stage macrocycle derivation at the site of ring closure is straightforward; the cyclic structure can be acylated, alkylated or subjected to native chemical ligation at the formed secondary amine to introduce diverse moieties (Figure 3c). Moreover, proper positioning of intramolecular imine traps in the form of tethered nucleophile groups (specifically, indoles and imidazoles, or thiols/selenols) leads to cyclized peptides with an embedded piperidine, and thia-/selenazolidine rings, respectively. These non-native structures in peptides can be easily oxidized, further augmenting rigidity of the macrocycle (Figure 3d).

Formation of (bi)cyclic peptides in vivo is far more challenging compared to chemical synthetic approaches due to the limited set of natural building blocks and incompatibility with reactive crosslinkers. Yet, Bionda et al. [40,41] have reported an innovative strategy for ribosomal synthesis of thioether-bridged peptide macrocycles in *Escherichia coli.* Here, intein-based protein splicing is combined with a chemoselective reaction between a cysteine and the genetically-encoded artificial amino acid *O*-(2-bromoethyl)-tyrosine (O2beY) that harbors a bromine-activated electrophile. Split-intein circular ligation of peptides and proteins method (SICLOPPS) takes advantage of intein splicing to generate cyclized peptides. Inteins are self-excising protein domains that link their consecutive sequences with a peptide bond, while liberating the intervening portions (exteins) as head-to-tail cyclized peptides [42]. Introduction of O2beY in the amino acid sequence flanked by intein sequences required engineering an aminoacyl-tRNA synthetase enzyme to charge this unnatural amino acid onto a tRNA recognizing the amber stop codon (see Section 3.6). In an amber suppressor *E. coli* strain, O2beY was thus incorporated into extein peptides, and the bromine-activated alkyl spontaneously reacted with the cysteine thiol group when positioned 2 to 8 residues apart (i.e., [i, i + 3] to [i, i + 9] arrangements), yielding bicyclic structures. Importantly, the electrophilic side chain of O2beY possesses the appropriate reactivity to engage with a proximal cysteine residue, while it does not interfere with any potentially competing nucleophiles in the cytoplasm [40].

### 3.2. Chemical Ligation of Peptides onto Scaffolds (CLIPS)

**Figure 4:**
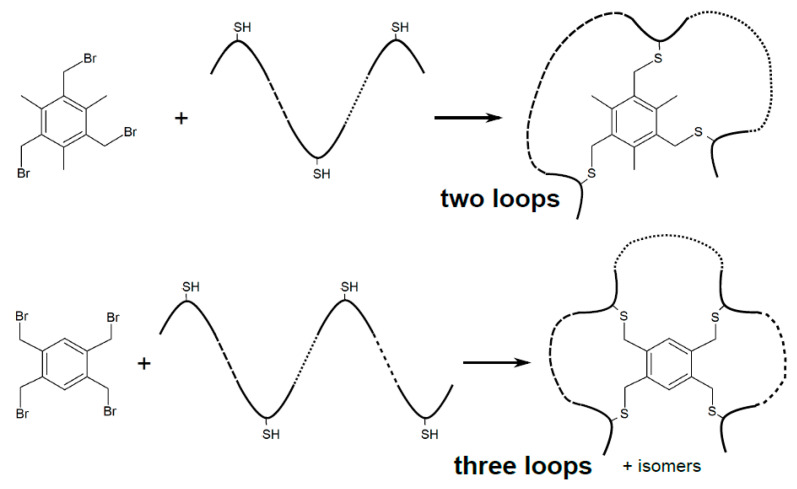
Single-step synthesis of double- and triple loop peptides, by treating cysteine-containing linear peptides with 2,4,6-tris(bromomethyl)mesitylene and 1,2,4,5-tetrabromodurene, respectively (adapted from [14]).

The CLIPS (chemical ligation of peptides onto scaffolds) technique for constructing peptide multicycles was initially described in a seminal paper by Timmerman et al. in 2005 [14]. Here, free cysteines in linear unprotected peptides selectively react in aqueous solutions with bromine-activated electrophiles of a planar aromatic scaffold, such as 2,4,6-tris(bromomethyl)mesitylene or 1,2,4,5-tetrabromodurene, to yield bi- or tricyclic peptide structures, respectively (Figure 4). Synthesis of a bicyclic structure requires three reactive groups on both, the peptide and the scaffold, and gives a single product, whereas four such reactive pairs are needed to produce a mixture of four three-looped regioisomer peptides. The mild reaction conditions and high selectivity of the alkylating reaction prompted Heinis et al. [43] to couple the technique with phage display for construction of chemically-constrained DNA-encoded peptides harboring three cysteines separated by two stretches of six randomized residues (i.e., the CX_6_CX_6_C format). This system simplifies screening of libraries made up of bicyclic peptide variants in excess of 10^9^ clones. Before being CLIPSed, the phage-displayed peptides were reduced with tris(2-carboxyethyl)phosphine to break the disulfide bonds.

**Figure 5:**
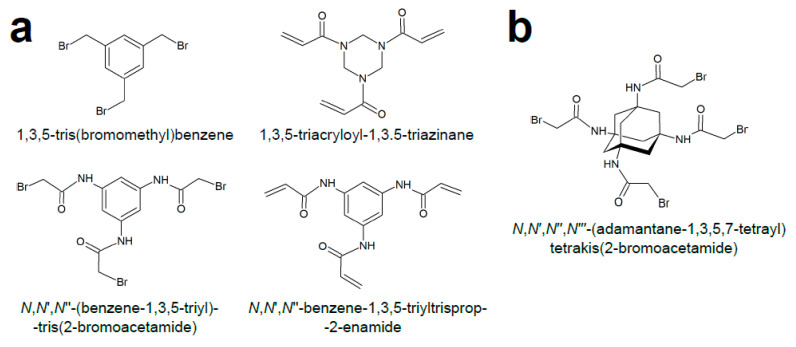
Structurally diverse symmetric CLIPS crosslinkers. (**a**). Examples of tridentate CLIPS crosslinkers imposing distinct peptide loop conformations [44]. (**b**). Tetradentate CLIPS crosslinker used by Ernst et al. [45] to construct a bicyclic peptide binder of the IgG Fc region. A biotin group was tethered via the fourth crosslinker arm to support immobilization of the peptide to paramagnetic streptavidin-coated beads for pull-down experiments.

**Figure 6:**
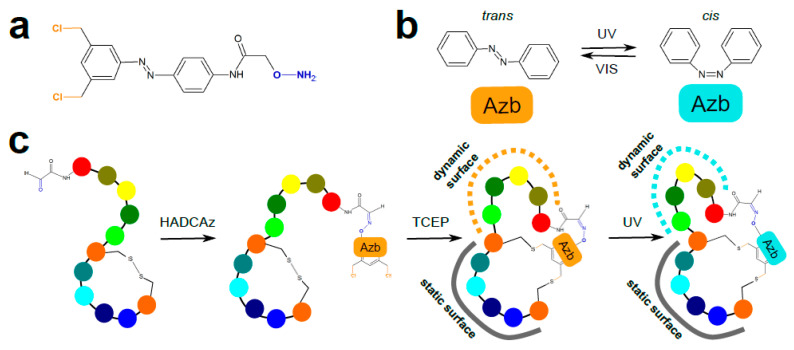
The use of azobenzene-based crosslinker to construct light-responsive bicyclic peptides (adapted from [46]). (**a**) A tridentate hydroxyl amine/dichlorobenzene-containing azobenzene (HADCAz) crosslinker. (**b**). Ultraviolet (UV) light-induced reversible geometric (configurational) isomerization of azobenzene (Azb). (**c**) Sequential bicyclization of N-terminally glyoxal-functionalized peptide with the HADCAz crosslinker. TCEP—reducing agent tris(2-carboxyethyl)phosphine used to break the disulfide bond.

Later, a number of different scaffolds (Figure 5a) have been developed to tie together three cysteine residues of an initially linear peptide [44] and have been demonstrated to exert a profound effect on the resulting bicyclic peptide conformation. This indicates that unprecedented structural diversity might be achieved by cyclizing a single peptide library with distinct alkylating scaffolds. Consequently, the likelihood of identifying hits by screening bioactive macrocycles with improved properties could be significantly increased. Further scaffolds have been designed to support two-step peptide modification (i.e., formation of a bicyclic peptide and subsequent functionalization of an additional reactive group [45] (Figure 5b). This allowed simple immobilization of conformationally constrained bicycles on solid matrices. In addition, Jafari et al. [46] have designed a scaffold that undergoes geometric isomerization when irradiated with UV light, leading to a conformational shift in one of the peptide loops (Figure 6). This feature might be harnessed for development of peptides with tunable binding affinity.

A similar cysteine crosslinking technique was developed by the Pentelute lab at MIT [47]. They are using hexafluorobenzene as a thiol-specific linker. The reaction proceeds with regioselectivity to 1,4-(para) disubstituted products, likely due to steric hindrance of the 2,5-(ortho) configuration, while the initially formed thioether moiety (the monosubstituted species) activates the aromatic carbon atom at position 4 for the second nucleophilic attack [48]. The nucleophilic aromatic substitution reactions (SNAr) take place at room temperature in presence of Tris base (in DMF), are highly selective for thiols and give near-quantitative peptide macrocycle yield [47]. Several perfluoroaromatic crosslinkers of different length (containing thioether moieties) can be used to modulate the macrocycle topology.

### 3.3. Enzymatic Peptide Cyclization

Enzymatic approaches complement synthetic chemical methods of peptide cyclization and offer certain advantages, such as chemoselectivity (both regio- and stereoselectivity). On the other hand, some of the enzymes display modest catalytic activity, rely on fairly large recognition motifs (hence leaving behind a trace of several defined residues in peptide macrocycles), catalyze competing reactions (such as hydrolysis or interchain peptide ligation), and most can only be applied to cyclization of polar (i.e., water-soluble) peptides (Table 1).

The first enzymatic head-to-tail circularization was achieved with sortase A [49], a transpeptidase from *Staphylococcus aureus*, implicated in anchoring surface proteins to the cell wall. The enzyme recognizes the C-terminal motif LPXTG (where X denotes any amino acid), cleaving it between the threonine and glycine residues and forming a thioester acyl-enzyme intermediate. The activated acyl donor is then transferred to an aminoglycine nucleophile within the pentaglycine cross-bridge of peptidoglycan. When peptides longer than 16 residues harboring N-terminal (oligo)glycine and C-terminal LPXTG recognition motif are subjected to sortase A treatment, the enzyme preferentially catalyzes intramolecular ligation resulting in a macrocycle [50]. However, the cyclization reaction is competed by oligomerization (especially with short peptides) and relinearization of already cycled peptides at thermodynamic equilibrium. In comparison to sortase A, asparaginyl endoprotease butelase 1 has only a minimal recognition motif and displays a much higher catalytic efficiency [51]. Butelase 1 is not amenable to recombinant expression and is produced by isolation from the legume plant *Clitoria ternatea*, where it catalyzes the cyclization of small disulfide-rich cyclotides. It recognizes a short C-terminal motif (N/D)HV and cleaves off the HV dipeptide. The asparagine/aspartate-ended peptide forms a thioester acyl-enzyme intermediate, and the activated carbonyl group is handed to the peptide’s N-terminus, hence rescuing the active site cysteine and forming a cyclized product. Butelase 1 is fairly promiscuous concerning the N-terminal sequence. It tolerates most amino acids (even in *D*-configuration; except for Asp, Glu or Pro) at position 1, and small hydrophobic residues (Leu, Ile and Val) or Cys at position 2, but has a strict requirement for *L*-Asn/*L*-Asp at C-terminus, thus leaving behind only a minimal footprint [52]. Further extension of the enzymatic cyclization toolkit came from redesigning *Bacillus sp.* serine endoprotease subtilisin’s active site [53]. The so-called peptiligases require carboxamidomethyl ester-activated C-terminus and catalyze peptide bond formation with the N-terminal amine without significant ester moiety hydrolysis [54]. These enzymes have broad substrate specificities (essentially allowing traceless cyclization) and extraordinary catalytic efficiency. They tolerate organic co-solvents (e.g., up to 50% DMSO or DMF) as well as denaturing agents (e.g., up to 2 M urea/guanidinium), thus being compatible with cyclization of hydrophobic peptides [54].

**Table 1:** Characteristics of enzymes used for peptide cyclization (adopted from [55]).

Enzyme	N-Terminal Recognition Sequence	C-Terminal Recognition Sequence (Cleavage Site Denoted with Caron Sign)	Ring Sizes (Residues)	Advantages	Disadvantages
sortase A	(G)_n_	LPXTˇG	≥16	commercially available, recombinant, high ligation yield	LPXT(G)_n_ footprint, reaction reversible, competing oligomerization, moderate catalytic efficiency (0.1–1 molar eq. required)
butelase 1	X_1_X_2_, where X_1_ is not P, D, or E, and X_2_ is L, I, V or C	(N/D)ˇHV	≥10	broad substrate specificity (thus minimal footprint), high ligation yield, high catalytic efficiency (~0.005 molar eq. required), reaction irreversible	only accessible through isolation, very few residues tolerated in position X_2_
peptiligases [omniligase]	any dipeptide lacking P	essentially any tetrapeptide without C-terminal P; requires activated C-terminal carboxy group (ester)	≥13	very broad substrate specificity (hence no footprint), extraordinary catalytic efficiency (only ~0.0003 molar eq. required), reaction irreversible, high yield, [tolerates co-solvents and chaotropic agents, commercially available, compatible with non-peptidic backbone moieties]	requires activated C-terminal carboxy group (ester)

**Figure 7:**
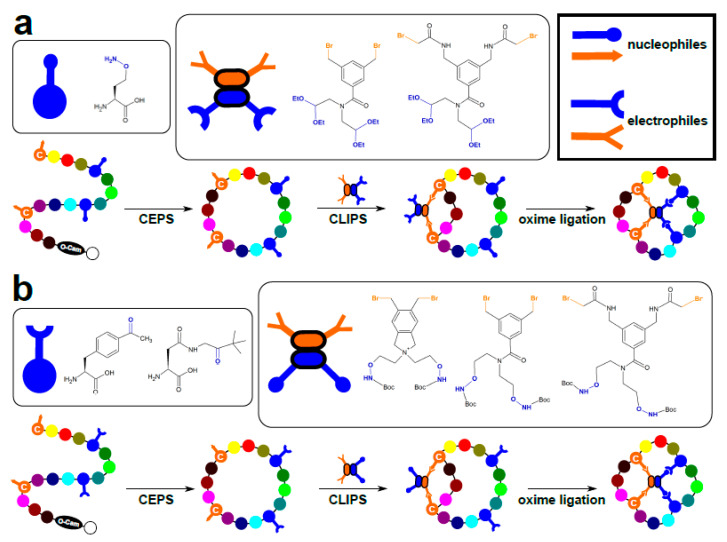
Three-stage macrocyclization of unprotected peptides, combining chemo-enzymatic peptide synthesis using omniligase-1 (CEPS), chemical ligation of peptides onto scaffolds (CLIPS), and oxime ligation (adapted from [58]). Two distinct strategies are depicted, differing in CLIPS crosslinkers and cognate residues harboring orthogonally reactive functional groups. (**a**). CLIPS with dibromo-/dicarbonyl crosslinkers, and aminooxy-homoserine residues. (**b**). CLIPS with dibromo-/di(aminooxy) crosslinkers and carbonyl group-containing residues. C—cysteine, *O*-Cam—carboxamidomethyl (Cam)-ester, Boc—tert-butyloxycarbonyl.

Recently, Schmidt et al. [56] reported omniligase 1, a new peptiligase that has since been made commercially available, compatible with chemo-enzymatic synthesis (CEPS). They combined enzymatic head-to-tail cyclization and CLIPS to produce tricyclic structures. The synthesis was quantitative only when CEPS preceded CLIPS, which can be attributed to the severely limited flexibility of the CLIPSed peptide backbone, reducing cyclization ligation efficiency. Omniligase 1 turned out to be a remarkably versatile tool, being capable of producing macrocycles containing non-peptidic moieties (e.g., polyethylene glycol spacers), isopeptide bonds (i.e., linkage of adjacent residues via amide bond of Lys ε-amino group), or *D*-amino acids provided they were located outside the enzyme recognition sites. The same research group used omniligase 1 in two additional studies. In the first, they accomplished a one-pot cyclization and oxidative folding of various cysteine-rich miniproteins (cyclotides and defensins) at a multi-gram scale [57]. In the second, regioselective synthesis of constrained tetracyclic peptides was achieved by taking advantage of orthogonal succeeding CEPS, CLIPS, and oxime ligations [58]. For oxime ligation (the condensation reaction between an aminooxy group and a carbonyl electrophile), the aminooxy group was introduced in the peptide itself using an artificial amino acid aminooxy-homoserine, while the protected carbonyl groups were instilled via tetradentate bromide CLIPS crosslinkers. Alternatively, keto-aminoacids were introduced during peptide synthesis, and later combined with aminooxy group-containing tetradentate bromide CLIPS crosslinkers (Figure 7). All reactions took place in aqueous media under mild conditions. A similar orthogonal synthetic strategy, termed ‘triple-C’ (for CEPS/CLIPS/CuAAC), combines omniligase 1-catalyzed head-to-tail cyclization, CLIPS, and the commonly used click-reaction of copper(I)-catalyzed azide-alkyne cycloaddition (CuAAC [19]).

### 3.4. Peptide Stapling

**Figure 8:**
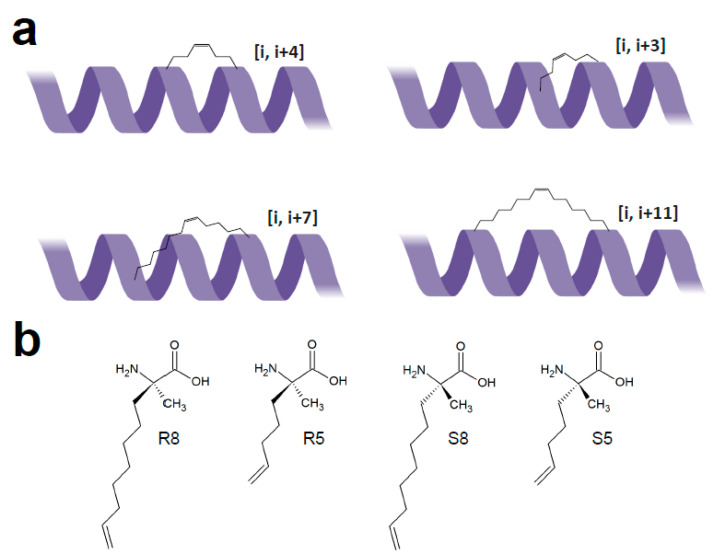
Peptide stapling configurations (adapted from [5]). (**a**). Common staple insertion positions. (**b**). Examples of designed alkene group-containing α,α-disubstituted amino acids used to introduce all-hydrocarbon staples into peptides via ring-closing metathesis. R and S refer to the stereo configuration, and numbers denote the length (carbon atom number) of olefinic side chains.

Protein–protein interactions (PPIs) often involve helical secondary structural elements of one protein partner binding to large flat or shallow surfaces of the other. Such binding sites are deemed undruggable with conventional small molecular weight compounds, hence helical peptides designed based on natural protein ligands show great potential as PPI inhibitors. However, their helical conformation in solution is typically not very stable in absence of full protein fold [5]. Thus, crosslinking of α-helix residues located opposite to the binding face (‘stapling’ in [i, i + 3], [i, i + 4], [i, i + 7] or [i, i + 11] arrangement) is required for imposing the desired (i.e., target binding) spatial configuration (Figure 8). Potency/affinity and/or cell permeability are thereby improved. Unfortunately, there is no general rule on choosing the optimal staple type, and laborious experimental optimization on a case-by-case basis is needed. The ‘stapling’ chemistry (Table 2) is similar to the crosslinking/bridging described above, but its efficiency is highly dependent on positioning, length, and stereochemistry of the staples. For synthetic peptides, unnatural amino acid residues harboring selectively reactive side-chain functional groups are incorporated at specific sites. Subsequently, the induced covalent bond locks the peptide to the helical conformation. Lactamization—intramolecular amide-bond formation between amino and carboxy group-containing residues (lysine or ornithine, and glutamate or aspartate, respectively)—is an example of crosslinking strategy allowing stapling of peptides composed of all-proteinogenic residues. However, it is limited to [i, i + 4] arrangement and requires additional orthogonal protecting groups for amino and carboxy functionalities that can be selectively removed prior to cyclization. On the other hand, cysteines positioned in a [i, i + 4] arrangement in unprotected peptides can be selectively stapled with hexafluorobenzene under mild conditions (see Section 3.2; [48]). Owing to the highly lipophilic character of the staple and its rigidity, the hexafluorobenzene-stabilized helical peptides demonstrated increased cell permeability in addition to improved protease resistance and binding affinity.

In addition to simple crosslinking of two side chains, double stapling or ‘stitching’ techniques were developed to further enforce helical peptide conformation. In stitching, two staples are introduced at [i, i + 4, i + 7] or [i, i + 4, I + 11] arrangement with the central residue i + 4 having two side chains (such as bis-pentenylglycine) that crosslink via ring-closing metathesis to the side chains of the proximal and distal residue [67]. Such reinforced peptides possessed higher helicity compared to the simpler [i, i + 7] stapled analog.

Staples are not necessarily just passive stabilizers of helical structures. Analysis of 52 X-ray structures for stapled helical peptide:protein complexes from the RCSB-PDB indicated that staple linkers interacted with the target protein surface in 17 cases, engaging in van der Waals and π–π interactions, and hydrogen bonding [5]. Furthermore, when the side chain linking is designed as a two-component reaction, the staple can be used as a reactive handle to append additional functionalities to helical peptides. These include fluorophore or biotin labels, and peptide ligation [66,68]. The stereochemistry of the staple introduced in a two-component reaction should not be underrated; for example, an in-tether chiral center strongly influenced the helical character, cell permeability, and binding affinity of stapled peptides [69,70]. Importantly, if two reactions of the same type are used to staple the peptide with an asymmetric crosslinker, this leads to diastereomer mixture formation. This problem can be solved by relying on two stepwise chemoselective reactions (e.g., copper(I)-catalyzed cycloaddition followed by ruthenium-catalyzed ring-closing metathesis) [68] to introduce a bifunctional chiral staple in a manner to produce a single diastereomer.

### 3.5. β-Hairpins and Hairpin Loops

β-hairpin motifs are another type of secondary structural elements commonly exploited by proteins for engaging in selective intermolecular interactions. Mimicking such structural motifs emerged as an innovative approach in drug discovery for addressing the ‘undruggable’ targets [6,71,72]. β-hairpins can be incredibly diverse due to differences in loop size, amino acid sequence variations, occurrence of β-bulges within the pairing antiparallel strands, as well as variations in the hairpin register [72]. The latter relates to potential ‘shifts’ of one strand relative to another, which determines the pairs of alternating cross-strand residues that occupy hydrogen-bonding positions rather than non-hydrogen-bonding ones. This, in turn, specifies which residue side chains are displayed on each of the two hairpin faces.

**Figure 9:**
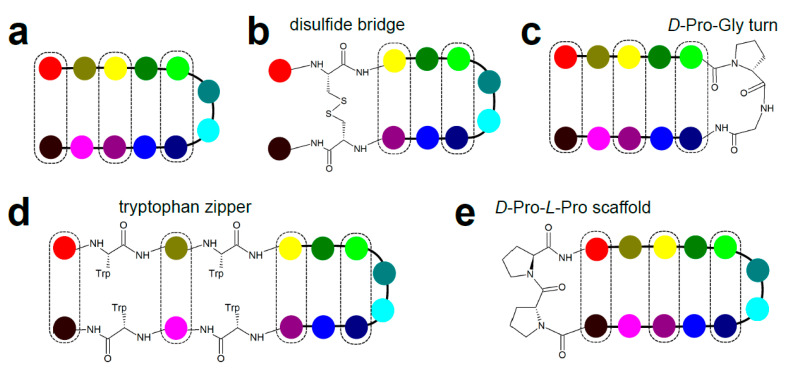
Strategies of stabilizing β-hairpin structures. (**a**) Schematic depiction of β-hairpin with residues at hydrogen-bonding positions highlighted in dashed boxes. (**b**) β-hairpin stabilized by an intramolecular disulfide bond. (**c**) β-hairpin promoting turn (e.g., *D*-Pro-Gly) to induce antiparallel β-sheet structure. (**d**) Tryptophan zipper stabilizing the β-hairpin via π–π interactions between cross-strand residues located at non-hydrogen-bonding positions. (**e**) Peptide grafting on the β-hairpin promoting *D*-Pro-*L*-Pro scaffold.

There are several ways of stabilizing the β-hairpin structure for a peptide taken out of the protein fold context (Figure 9). The simplest is to flank the β-hairpin motif with two cysteines and form a disulfide bridge [73]. Cystine-based macrocycles are, however, prone to reductive opening and associated with rather high flexibility due to significant rotational freedom of disulfide bond. Another approach relies on edge-to-face π–π stacking interactions between the cross-strand tryptophan residues located at non-hydrogen-bonding positions (called the tryptophan zipper motifs) to stabilize the antiparallel β-strand pairing [74,75]. Both rigidification strategies are compatible with biological peptide display libraries as they depend on proteinogenic amino acid residues as stabilizers. Conversely, attempts to impose a β-hairpin conformation by optimizing the turn region are mainly applicable to synthetic peptides as non-proteinogenic residues typically need to be incorporated into linear peptides. Here, dipeptide motifs, such as Asn-Gly [76], *D*-Pro-Gly [77], Aib (α-aminoisobutyric acid)-Gly [78], or Aib-*D*-Pro [79,80] were reported as nuclei promoting β-hairpin conformation by assuming the type-I’ or type-II’ turn structure. These turn types supposedly induce the preferred right-handed twist of a hairpin [72]. Finally, grafting the peptide loop on a scaffold that promotes the antiparallel strand pairing is a popular method of β-hairpin induction. The dipeptide *D*-Pro-*L*-Pro is a well-characterized β-hairpin-promoting scaffold adopting a type-II’ turn onto which termini of a β-hairpin peptide can be ligated [81,82,83]. The pair of ‘terminal’ residues now positioned across (directly attached to this template) becomes a hydrogen-bonding pair [72], giving rise to an in-register antiparallel β-sheet mimetic.

Efficient pairing of short antiparallel β-strands provides access to looped peptides of β-sheet-loop-β-sheet topology. Based on sequence analysis of 8-residue hairpins found in proteins, Ramirez-Alvarado et al. [76] designed a small peptide named BH8 (with the core sequence Ile-Thr-Val-Asn-Gly-Lys-Thr-Tyr) that formed a fairly stable hairpin conformation in water. In BH8, the central Asn-Gly dipeptide assumes a type-I’ turn, while threonine residues located at non-hydrogen-bonding positions display high tendency for β-sheet formation. The rest of the residues were chosen based on the occurring frequency in natural hairpins. Building on this minimal β-hairpin motif, Sawada et al. [84] created a phage-displayed hairpin loop library by exchanging the turn-inducing Asn-Gly dipeptide with a stretch of four or five random amino acids. To reinforce the loop stability, they added terminal lysine and glutamate residues for salt bridge formation. The library was panned against insulin, yielding a number of diverse sequences. Hit structural analysis confirmed the β-turn and antiparallel β-sheet content. Interestingly, in several ligands, two cysteine residues were present in the loop region, reinforcing the β-hairpin loop structures by intramolecular disulfide bonds. This was associated with greater association rate constants of such peptides, indicating that rigidness is a prerequisite for fast target binding.

Also starting from the salt bridge-stabilized ‘consensus’ hairpin peptide BH8, Pastor et al. [85] randomized all four residues at hydrogen-bonding positions to construct a library of ~140,000 peptides and screened it for β-hairpin structures. Several new peptides were identified, suggesting that systematic combinatorial approaches are a powerful tool for structure optimization as cooperative interactions clearly developed within the randomized cluster of residues. In fact, two of the peptides (having the core sequences Trp-Thr-Tyr-Asn-Gly-Ile-Thr-Tyr and Tyr-Thr-Tyr-Asn-Gly-Ile-Thr-Tyr; the residues differing from BH8 sequence are underlined) displayed significantly higher β-hairpin character in solution compared to the parent peptide (66 ± 4% and 69 ± 4%, respectively, vs. 24 ± 6%).

### 3.6. In Vitro Molecular Evolution

De novo design of polypeptides with defined tertiary structures pre-organized to bind selected targets remains challenging even for the shortest peptides [86,87]. Mimicking evolutionary principles of exerting selection pressure at large collections of randomly created variants is thus a convenient strategy for developing structures with pre-defined functions [88]. Introducing distinct structural elements capable of forming crosslinks at specific positions of otherwise randomized peptides confers a degree of rigidity to all library elements. Even though the vast majority of them do not possess appropriate sequence/structure for a given function, screening will likely uncover the few that do, providing that the library complexity (i.e., diversity) is high-enough. Synthetic peptide libraries are typically limited in size and are only amenable to single-round screening (e.g., sorting the combinatorial one-bead-one-compound libraries by means of continuous-flow techniques, and identifying the hits by mass spectrometry). Conversely, molecular evolution is possible when the library entities are capable of replicating. In this way, selection pressure exerted at succeeding generations facilitates enrichment of fittest binders and minimizes background (i.e., non-specifically retained elements). In biological libraries, peptides are encoded by nucleic acids, which are amenable to replication, both in vivo (in cells) and in vitro (using polymerase chain reaction). The crucial feature of genetically encoded libraries is thus a direct link between the genotype (the encoding nucleic acid) and the phenotype (the cognate encoded peptide). Many different platforms, such as phage display, bacterial display and mRNA display, have been developed for the purpose of screening vast collections of peptides (reviewed in [88]), and will not be discussed here in detail. Although biological peptide libraries are restricted to proteinogenic amino acids, additional synthetic approaches, genetic code manipulation and/or (semi)rational template design is also compatible with constrained peptide structures.

**Figure 10:**
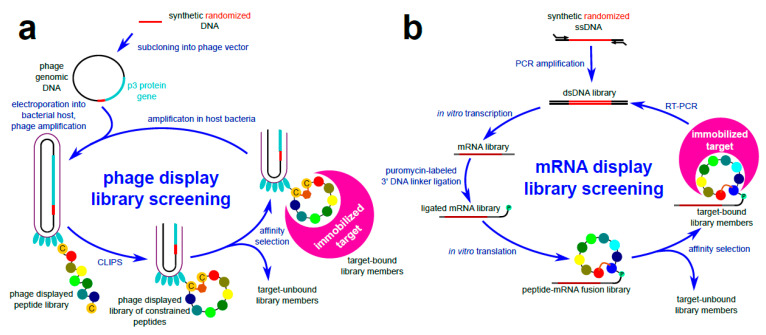
Schematic depiction of phage display (**a**) and mRNA display (**b**) library screening procedures. (**a**). Phage-displayed peptide library is constructed by subcloning randomized oligonucleotides in a phage vector in frame with p3 minor coat protein, leading to peptide-p3 encoding fusion genes. The resulting DNA library is electroporated into host bacteria where phage virions are assembled. Phages are isolated from bacterial culture. If the displayed peptides were designed to harbor paired cysteine residues (C), this feature can be exploited for macrocyclization via chemical ligation onto scaffolds (CLIPS; Section 3.2). Phage library is incubated with an immobilized target protein. Target-unbound virions are removed by washing, while the bound ones are amplified by infecting bacterial host for further selection rounds. (**b**). In mRNA display, the encoding DNA library is PCR-amplified from randomized synthetic oligonucleotides and in vitro transcribed to mRNA library. Next, puromycin (P)-labeled DNA linker is ligated at the 3′ end of mRNAs and the ligated mRNA library is translated using a cell-free system. Genetic code expansion and/or reprogramming can be used to introduce non-proteinogenic residues for peptide cyclization (see Figure 11). During nascent peptide synthesis, puromycin enters the A site of the ribosome (not shown) and forms a covalent link between the transcript and the displayed peptide. The peptide-mRNA fusion library is then subjected to affinity selection against an immobilized target protein. Washing steps remove target-unbound clones, while retained library members are reverse-transcribed and PCR amplified for further selection rounds. Note the direct genotype–phenotype linkage in both platforms, required for peptide identification via DNA sequencing.

As discussed above, one way to construct a library of constrained linear peptides is to flank the randomized sequence with short stretches of residues with the propensity to assume antiparallel β-strand fold, thus forming hairpins [84]. These structures rely on weak hydrogen bonds for conformational rigidification and are not very stable in solution. Another simple strategy is to introduce paired cysteine residues in peptide chains, thereby affording cyclization via disulfide bonds. In phage display (Figure 10a), a technique of expressing foreign (poly)peptides tethered to bacterial virus capsid via fusions with structural proteins, the disulfide bond is formed concomitantly with viral assembly in oxidative periplasm of host bacteria. Conversely, bicyclic peptide libraries can be displayed on phage if three-cysteine-containing peptides are crosslinked with trifunctional alkylating scaffolds (see Section 3.2), yielding single isomers [55]. However, the technique of alkylating reduced thiol groups directly on phage virions is rather technically demanding [89], and the macrocycle topology is pre-defined with specific positioning of the cysteine residues within the otherwise randomized peptide sequence. It is well documented that the display of peptides on filamentous phage capsid is biased against the odd number of cysteines. Indeed, the infectivity of phages displaying three-cysteine-peptides (to be tethered to a scaffolding unit) was two orders of magnitude lower compared to that of a wild-type phage, necessitating phage amplification in large host bacteria culture volumes [55]. Chen et al. [90,91] have designed phage-displayed peptide libraries of the X_o_CX_m_CX_n_CX_p_ format (where X is any amino acid residue, *o*, *p* = 0 or 1, and *m*, *n* = 3–6). They showed that in affinity selected peptides a fourth cysteine is commonly present in one of the randomized regions, forming a second disulfide bond. Importantly, this strategy allowed for a high number of different bicycle topologies (as many as 3 × (o + m + n + p)), which (combined with randomized stretches of peptide) drastically widens the structural space of bicycles. In mRNA display, where the peptides are produced using in vitro translation (and hence do not enter specialized cell compartments), disulfide cyclization may not be straightforward. However, free paired cysteines can still be crosslinked with a bromine-activated dialkylating agent (see Section 3.2) [92].

**Figure 11:**
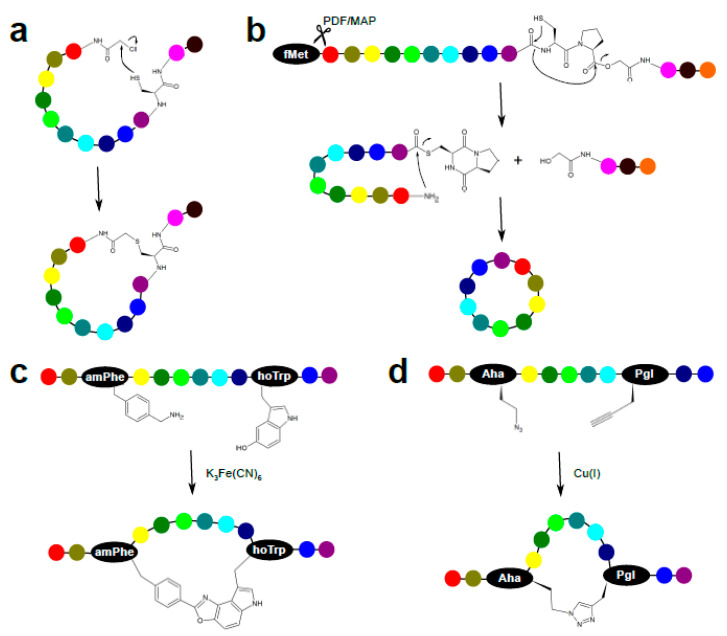
Examples of intramolecular macrocyclization reactions achieved through incorporation of designed non-natural residues in peptides (adapted from [4]). (**a**) Reaction of a chloroacetylated N-terminus with a cysteine residue to yield a thioether bond. (**b**) Seamless amide cyclization via enzymatic liberation of the amino moiety at the N-terminus (PDF—protein deformylase, MAP—methionine aminopeptidase), followed by spontaneous rearrangement of a cysteinyl-prolyl-glycolic acid sequence. (**c**) Hydroxyindole- and benzylamine-mediated oxidative coupling. (**d**) Copper(I)-catalyzed azide-alkyne cycloaddition. amPhe—aminomethyl phenylalanine, Aha—azidohomoalanine, fMet—N-formylmethionine, hoTrp—hydroxytryptophan, Pgl—propargylglycine.

In phage display, virion’s capsid to which a specific peptide is tethered encapsulates viral genomic DNA harboring the fusion gene encoding that peptide. In mRNA display (Figure 10b), the phenotype-genotype link is provided through the formation of a covalent bond between the puromycin-coupled 3′ end of an mRNA molecule and the C-terminal amino acid residue of the peptide it encodes. Following affinity panning, the retained peptide-mRNA conjugates are reverse transcribed, PCR-amplified, and subjected to in vitro transcription and puromycin ligation. The puromycin moiety (mimicking charged tRNA) enters the A site of ribosomes and is ligated to the nascent peptide, terminating the translation process. Cell-free translation systems are used for generating peptide-mRNA conjugates entering subsequent selection rounds. Not depending on host cell transcription/translation mechanisms, mRNA display has three major advantages over phage display. First, the low efficiency of bacterial transformation severely limits the size of phage display libraries. Thus, complexities of mRNA display libraries surpass those of phage display by up to five orders of magnitude. Second, in phage display, coat-tethered peptides need to cross the bacterial plasma membrane, which is reflected on library bias toward better translocating peptide sequences. Third, reliance on cell-free translation systems allows for genetic code expansion or reprogramming, and thereby ribosomal incorporation of non-proteinogenic amino acid containing non-canonical functional groups that can be exploited for crosslinking and macrocycle formation (reviewed in [4]). Genetic code expansion relates to chemoenzymatic charging (using an orthogonal aminoacyl-tRNA synthetase (ARS) [93]) of a tRNA. To introduce a non-proteinogenic or unnatural amino acid into the genetic code, a novel tRNA-ARS pair is formed. A stop codon (or a four-base codon) is assigned and used by this orthogonal set, widening the genetic code to 21 amino acids. Conversely, in genetic code reprogramming, cell-free translation systems composed entirely of purified components (called PURE for Protein synthesis Using Recombinant Elements) are used. PURE systems may lack specific natural release factors and ARSs, and contain orthogonal ARS-non-canonical amino acid pairs. The degenerate nature of the genetic code enables researchers to tamper with and reserve redundant codons for specific non-proteinogenic amino acids. Examples of peptide macrocycles accessible through genetic code reprogramming and compatible with mRNA display are shown in Figure 11. The mRNA display and genetic manipulation techniques are, however, technically demanding and therefore limited to a handful of specialized labs.

Cell-free translation systems (as applied in mRNA display) can be exploited not only to drastically widen the peptides’ chemical space by incorporation of non-proteinogenic/non-amino acid residues, but also by tampering with peptide topology. For example, it was observed that reprogramming the start codon to initiate transcription with an N-chloroacetylated α-amino acid (Figure 11a) leads to posttranslational peptide cyclization with the nearest upstream cysteine residue when there are two or more cysteines present [94]. Thus, a lariat-shaped structure with a small N-terminal cycle and a C-terminal tail with free downstream cysteine(s) is formed as a major product. On the other hand, installing a structurally constrained chloroacyl-containing initiator residue (e.g., N-chloroacyl-γCp-Phe dipeptide, where γCp is a cyclopropane-containing γ-amino acid) preferentially affords large peptide macrocycles [95]. Head-to-head comparison of screening experiments using mRNA-displayed peptide libraries differing in initiator residues confirmed that distinct cyclic peptides are enriched against the same molecular target [95]. Moreover, regioselective cyclization with distal cysteine residues may be exploited for subsequent CLIPSing of free cysteines, allowing bridging of cyclized peptides.

## 4. Applications of Constrained Peptides

In this last section, we discuss the development and diverse applications of constrained peptides. The review does by no means attempt to be exhaustive. Rather, we try to illustrate the exceptional properties of structurally pre-organized rigid peptides by limiting the discussion to a small number of representative studies.

Antimicrobial agents are traditionally associated with small-molecule chemistry. The development of novel peptidomimetic technologies, however, has steered the field towards macrocycles. ‘t Hart et al. [18] reported the use of a 1,3,5-tris(bromomethyl)benzene-CLIPSed bicyclic peptide phage display screen as an avenue to generating novel antimicrobial lipopeptides. Specifically, they have identified unique lipid II-binding peptides that are active against Gram-positive bacteria, including clinically relevant vancomycin-resistant strains. Another similar example came from Adaligil and coworkers [17]; using ’mirror image’ phage display (screening against enantiomers of *D*-alanyl-*D*-alanine and *D*-alanyl-*D*-lactate containing fragments of bacterial cell wall precursors and their structural mimetic cephalosporin, and subsequent *D*-amino acid peptide synthesis) has led them to bicyclic peptides composed entirely of *D*-amino acids. Two of the hits displayed relatively high antibacterial (including against methicillin-resistant *S. aureus* and vancomycin-resistant *Enterococci*), but no hemolytic activity. Related to antimicrobials, neutralization of bacterial endotoxins is a warranted medicinal application but has hampered small molecule use due to the large contact area of the interacting molecules in the septic shock cascade. By interrogating a synthetic β-hairpin peptide library, Gonzalez-Navarro et al. [96] identified novel peptide binders with LPS neutralizing activity. Furthermore, Srinivas et al. [97] have identified *D*-Pro-*L*-Pro template-stabilized β hairpin mimetics of the antimicrobial peptide protegrin I, highly active against *Pseudomonas aeruginosa*. Using an elegant approach in which forward genetic screening was combined with functional and biochemical assays they have identified the outer membrane protein LptD as the molecular target for the peptidomimetic antimicrobials. It was concluded that perturbation of the critical LPS transport function of LptD is responsible for bacterial cell death, paving the way to antimicrobials with novel mode of action.

Novel peptidic technologies also support efficient protein–protein interaction probing. In their pioneering work, Timmerman et al. [14] have demonstrated the value of chemically crosslinked peptides in discontinuous epitope mapping. They have constructed a synthetic library of dodecamer peptides tiled along the sequence of follicle-stimulating hormone (FSH) β chain, both linear and cyclized, and analyzed binding to a monoclonal antibody using ELISA assay. Interaction was only detected with a specific looped peptide, and systematic substitutions allowed the identification of residues constituting the antigenic determinant (the β3-loop). In a follow-on paper [98], immunological properties of cyclized peptide mimetics of the β3-loop were studied. When rats were immunized with the 1,3-bis(bromomethyl)benzene-looped peptide (as opposed to the disulfide-stabilized counterpart), antibodies selectively recognizing the β subunit of FSH were induced. Double stabilization (involving an additional disulfide bond at the loop termini) further augmented the FSH-targeted immunogenicity, clearly demonstrating the importance of subtle conformational differences for inducing cross-reactive antibodies. The successful mimicry of a complex protein surface by a small synthetic peptide attained by CLIPS technology indicates its enormous potential for generating antibodies against difficult protein targets such as integral membrane proteins, where immunization with conventional linear peptides does not deliver results. Iqbal et al. [99] devised a high-throughput strategy for hydrocarbon-stapled peptide identification using mRNA display. They reprogrammed the genetic code to incorporate α-methyl cysteine at positions i and i + 4 of short randomized peptides using the PURE system, and stapled them with m-dibromoxylene. Although only a proof of principle screen was conducted with the library, this approach has the potential of evolving into a powerful tool for PPI inhibitor discovery.

Constrained peptides are being increasingly applied in oncology. For example, by screening a phage display library, Anananuchatkul et al. [100] identified [i, i + 7]-stapled helical peptides that showed a disruptive ability for the hDM2 oncogene-p53 tumor suppressor interaction. A similar approach has yielded binders for galectin-3 (a cancer-related galactose-binding protein) from a stapled α-helix phage-displayed peptide library [101]. Bertoldo et al. [15] reported a novel binder, identified by a screening bicyclic phage display peptide library, to the interaction region of the translational Wnt inhibitor ICAT (inhibitor of b-catenin and Tcf), which is a prime target site on β-catenin for therapeutic intervention in oncology. By screening libraries of CLIPSed bicyclic peptides designed on the basis of anti-gastrin 17 antibody complementarity determining loops, Timmerman et al. [102] have developed gastrin 17-neutralizing peptidomimetics. As gastrin 17 is a trophic factor in several gastrointestinal tumors, its neutralization presents a warranted anticancer therapeutic strategy. Indeed, the constrained peptides effectively reduced proliferation of two cancer cell lines in vitro, comparably with the parent neutralizing antibodies. The company Bicycle Therapeutics has a large portfolio of conformationally constrained macrocyclic peptides developed through phage display and CLIPS technology, mainly for treatment of cancers. Several of their drug candidates rely on such peptide moieties for selective cytotoxic payload targeting [103,104,105] and some have already entered clinical evaluation (clinical study identification codes NCT03486730, NCT04180371, NCT04561362). Others are structured as multiple bicyclic peptides connected by linkers via a central hinge and target and activate CD137 (a co-stimulatory immune checkpoint molecule) on NK and T cells or (combining two different bicycles) simultaneously activate CD137 and target tumor-associated antigens [105]. An elegant approach utilizing bicyclic peptides for targeting intracellular PPIs was devised by Trinh et al. [20], where one of the rings was an invariant cell-penetrating peptide and the other contained a randomized peptide sequence. The library was screened against the oncoprotein K-Ras G12V and yielded a K-Ras inhibitor of moderate potency that disrupted signaling events downstream of Ras, and induced apoptosis of cancer cells. An identical approach was employed in the discovery of a macrocyclic peptide disruptor of NEMO-IKKβ interaction, inhibiting proliferation of cisplatin-resistant ovarian cancer cells [21]. A related principle was adopted by Bernhagen et al. [106,107]; they have screened bicyclic libraries with built-in universal integrin-binding sequence Arg-Gly-Asp in the first loop and a randomized tripeptide sequence in the second loop. With the ‘guided’ library, they have probed adjacent binding sites on α_5_β_1_, and identified high-affinity integrin binders with potential as therapeutics or delivery vehicles in diagnostics and treatment of breast cancer. Furthermore, it has been demonstrated that it is possible to mimic RNA-recognition motifs of natural proteins [26]. Encouraged by this finding, Shortridge et al. [27] have screened a cyclic β-hairpin peptide library designed to mimic bovine immunodeficiency virus trans-activator of transcription (Tat) RNA-binding domain against the primary miRNA-21 transcript’s stem loop using the electrophoretic mobility shift assay. A structured peptide was identified that bound to an oncogenic microRNA-21 precursor with decent affinity and specificity, and suppressed Dicer processing, preventing downregulation of key tumor-suppressing and proapoptotic factors.

Constrained peptides with PPI-disruptive activities are also being developed for combating inflammatory disorders. Tamada et al. [108] have designed a cyclic β-hairpin peptide mimicking the predicted receptor for advanced glycation end-products (RAGE)-binding domain of high mobility group box 1 (HMGB1) protein using in silico methods. As HMGB1, which is secreted from immune and dying cells during cellular infection and injury, is a major promoter of inflammation, the peptide blocking HMGB1/RAGE interaction could become a useful therapeutic against HMGB1/RAGE-mediated sepsis and other inflammatory diseases. Another study identified disulfide-cyclized peptide binders of TNF-α via phage display and utilized them as imaging contrast agents to inflammatory areas by covalently linking them to iron oxide nanoparticles [109].

Another exciting research area involving structured peptides is the development of minimalist peptide catalysts in which the residues are spatially arranged to mimic enzymes’ active sites in terms of functional group cooperativity in catalysis. Because de novo design of catalytic peptides would be highly challenging, screening libraries of partially randomized peptides with the propensity to fold into defined structures is a viable alternative. For example, both helical [110] and β-hairpin loop scaffolds [111,112] have been successfully exploited to nucleate active site-like residue organization with the imidazole ring of a central histidine serving as a nucleophile in acyl-transfer catalysis. Bezer et al. [110] designed polyalanine/aminoisobutyric acid-based helical peptide libraries with His at position i, and randomized residues at positions in the immediate vicinity (i.e., [i − 4], [i − 3], [i + 3], or [i + 4]). Similarly, Matsumoto et al. [111,112] designed synthetic peptide libraries by randomizing the non-hydrogen-bonding positions of a histidine-harboring β-hairpin loop peptide. Here, the effects of various aromatic and hydrogen-bonding residues in close contact with the histidine (its ±2 neighbors in the linear sequence and those located cross-strand) were probed. Dye-labeled substrates (active esters) were incubated with one-bead-one-compound peptide libraries in dichloromethane. In this reactive tagging assay, acyl groups were transferred on the imidazole group, forming colored N-acyl histidine intermediates stable under nucleophile-free conditions (whereas in presence of competing alcoholic nucleophiles the peptides were deacylated, thus closing the catalytic transesterification cycle). The intensely stained beads were then separated from the non-stained ones, and the peptide catalysts were identified by mass spectrometry. The authors note that both, the type of cooperating residues (enhancing imidazole’s nucleophilic character) and the structural determinants of the peptide (e.g., helix length and type (α vs. 3_10_), β-hairpin flexibility, and placement of functionalities within the structure), govern catalytic activity in a way that would be difficult to predict. These observations argue in favor of interrogating combinatorial peptide libraries for functional properties other than binding affinity.

## 5. Concluding Remarks

Conventional low-molecular-weight drugs are well-suited as bioactive ligands when it comes to docking into small clefts and pockets on target proteins, but fail to address large flat surfaces participating in protein–protein interactions. Small molecule drugs are also incapable of selectively binding difficult targets, such as small compounds and specific nucleic acid sequences/structures. The comparatively larger size of peptides and their immense structural diversity, on the other hand, are perfect for such applications. However, the inherent flexibility which undermines binding affinity, susceptibility to proteolytic enzymes, and poor cell penetration are properties severely hampering therapeutic use of linear peptides. Innovative strategies for peptide chain cyclization or crosslinking have been developed with the aim of restricting conformational flexibility (e.g., stabilizing the target-bound conformation), enhancing peptide metabolic stability, and/or improving cell permeability.

As rational design of peptide structures with desired functional properties is next to impossible, the combinatorial nature of peptides is exploited to construct and screen vast libraries of variants. Both synthetic and genetically-encoded library formats are amenable to structure rigidification, often allowing identification of bioactive peptides requiring no subsequent optimization. With ever-progressing synthetic routes and approaches, constrained peptides are destined to fill the untapped gap between the conventional small synthetic drugs and the more complex protein biologics. Looking at the bigger picture, constrained peptides are gaining popularity as tools in biomedical research (e.g., for target validation [113] or as pharmacophore models [108,114,115]) and as minimalist catalysts in organic synthesis [110,111,112].

## Figures and Tables

**Table 2:** Examples of common stapling chemistries to impose helical peptide conformation.

Chemistry	Residues Involved	Compatible Arrangement	Does Stereochemistry of the Staple Handles Need to be Considered?	Comment
alkene ring-closing metathesis	(homo)serine *O*-allyl ethers [59]	[i, i + 4]	no	all hydrocarbon staple
	α,α-disubstituted residues with olefinic side chains (R or S configuration, 5 or 8 atoms long) [60]	[i, i + 4] and [i, i + 7]	yes (S5/S5 for [i, i + 4], S8/R5 or S5/R8 for [i, i + 7])	
lactamisation	lysine and glutamate, or ornithine and aspartate [61]	only compatible with [i, i + 4] arrangement	no	requires extra orthogonal protective groups for amino and carboxy groups for on resin lactamisation
cycloadditions	azide and alkyne group containing residues with 4 + 2 or 4 + 3 methylene units long side chains [62]	[i, i + 4]	no	well-established click reaction (Cu(I)-catalyzed azide-alkyne cycloaddition)
	tetrazole and alkene group containing residues [63]			UV-induced cycloaddition between tetrazoles and alkenes to yield fluorescent pyrazoline tethers
disulfide bridges	thiol group containing residues [64]	[i, i + 7]	yes (combination of *D* and *L*-residues)	chronologically the oldest technique, requires acetamidomethyl protecting groups for thiols, staple unstable (prone to reduction)
thioether bridges	cysteine and an alpha-bromo amide group containing residue [65]	[i, i + 3] and [i, i + 4]	no	staple stable, higher helicity achieved with [i, i + 3] arrangement
	two (homo)cysteines + dichloroacetone crosslinker [66]	[i, i + 4]	no	bis-alkylating crosslinker amenable to further derivation via oxime ligation (e.g., fluorophore or biotin coupling)
	cysteines + perfluoroaromatic crosslinker (e.g., hexafluorobenzene) [48]	[i, i + 4]	no	regioselective reaction (para-disubstituted staple) proceeding under mild conditions in high yield even for unprotected peptides
